# Critical involvement of Rho GTPase activity in the efficient transplantation of neural stem cells into the injured spinal cord

**DOI:** 10.1186/1756-6606-2-37

**Published:** 2009-11-28

**Authors:** Fujiki Numano, Akihiro Inoue, Mitsuhiro Enomoto, Kenichi Shinomiya, Atsushi Okawa, Shigeo Okabe

**Affiliations:** 1 Department of Cell Biology, School of Medicine, Tokyo Medical and Dental University, 1-5-45 Yushima Bunkyo-ku, Tokyo 113-8519, Japan; 2 Department of Orthopaedic and Spinal Surgery, Graduate School, Tokyo Medical and Dental University, 1-5-45 Yushima Bunkyo-ku, Tokyo 113-8519, Japan; 3 Developmental Division of Advanced Orthopaedic Therapeutics, Graduate School, Tokyo Medical and Dental University, Bunkyo-ku, Tokyo 113-8519, Japan; 4 Department of Cellular Neurobiology, Graduate School of Medicine, University of Tokyo, Bunkyo-ku, Tokyo 113-0033, Japan

## Abstract

**Background:**

Transplantation of neural stem/progenitor cells is a promising approach toward functional restoration of the damaged neural tissue, but the injured spinal cord has been shown to be an adverse environment for the survival, migration, and differentiation of the donor cells. To improve the efficiency of cell replacement therapy, cell autonomous factors in the donor cells should be optimized. In light of recent findings that Rho family GTPases regulate stem cell functions, genetic manipulation of Rho GTPases can potentially control phenotypes of transplanted cells. Therefore we expressed mutant forms of Rho GTPases, Rac, Rho, and Cdc42, in the neural stem/progenitor cells and examined their survival and migration after transplantation.

**Results:**

Manipulation of the individual Rho GTPases showed differential effects on survival, with little variation in their migratory route and predominant differentiation into the oligodendroglial lineage. Combined suppression of both Rac and Rho activity had a prominent effect on promoting survival, consistent with its highly protective effect on drug-induced apoptosis in culture.

**Conclusion:**

Manipulation of Rac and Rho activities fully rescued suppression of cell survival induced by the spinal cord injury. Our results indicate that precise regulation of cell autonomous factors within the donor cells can ameliorate the detrimental environment created by the injury.

## Background

Neural stem/progenitor cells (NSPCs) are widely present in the developing mammalian central nervous system (CNS) and known for their capability of self-renewal and potential of differentiation into multicellular lineages [1,2]. The protocols of in vitro differentiation and maintenance of NSPCs have been established [3,4], and transplantation of NSPCs is thought to be an important approach toward functional restoration of the damaged CNS tissue, including injured spinal cords [5-7]. Previous studies have shown partial functional improvement after spinal cord injury by transplantation of NSPCs derived from the embryonic CNS or embryonic stem cells [8-11]. Although these results suggested the potential of NSPC transplantation in the improvement of spinal cord function, there have been a small number of reports on basic mechanisms of NSPC survival in the host spinal cord environment. Previous studies have shown that the injured spinal cord is not a favorable environment for the survival, migration, and differentiation of the donor cells [12,13]. Therefore, identification of regulatory mechanisms of NSPC survival and differentiation both in the intact and injured spinal cord environment should be important.

The Rho family small GTPases, members of the Ras superfamily, are known to regulate cell shape, movement, and adhesion in multiple mammalian cell types [14]. Rho GTPases also have been demonstrated to activate a number of signal transduction pathways involved in cell cycle progression, gene expression, and cell survival [15]. In the context of development and maintenance of neurons and glial cells in the CNS, negative roles of Rho family GTPases in cell survival have been implicated. For example, Rac/Cdc42 GTPases promote the apoptotic death of NGF-deprived sympathetic neurons [16,17] and activation of Rac by p75 neurotrophin receptor (p75^NTR^) induces apoptosis via activation of c-jun N-terminal kinase (JNK) in oligodendrocytes [18]. Rho activation is prominently enhanced in the injured spinal cord and involved in p75^NTR^-dependent apoptosis [19]. These experimental evidences suggest negative roles of Rho GTPase in survival of transplanted NSPCs in an adverse microenvironment of the injured spinal cord.

In this study we identified Rho and Rac GTPase activity as a potent regulator of cell survival after NSPC transplantation. Recombinant adenovirus-mediated expression of dominant-negative forms of RhoA and Rac1 increased the survival of transplanted cells more then two-fold in the intact spinal cord. Furthermore, expression of RhoDN and RacDN in NSPCs fully rescued down-regulation of cell survival after the transection of spinal cords. Taken together with strong protective effects of the same genetic manipulation against chemically induced apoptosis in vitro, these results indicate that Rho GTPase is one of the critical cell- autonomous factors promoting successful integration and survival of NSPCs in the injured spinal cord.

## Results

### Suppression of NSPC survival in the injured spinal cord environment

Our previous study of NSPC transplantation into the intact spinal cord indicated advantage of using hippocampus-derived NSPCs as a donor source in comparison with spinal cord-derived NSPCs [20]. Additionally, hippocampus-derived NSPCs showed more preferential migration toward the white matter of the host spinal cord. This preferential association with the white matter and directed differentiation of NSPCs toward the oligodendroglial lineage are advantageous in facilitating remyelination in the injured spinal cord.

**Figure 1:**
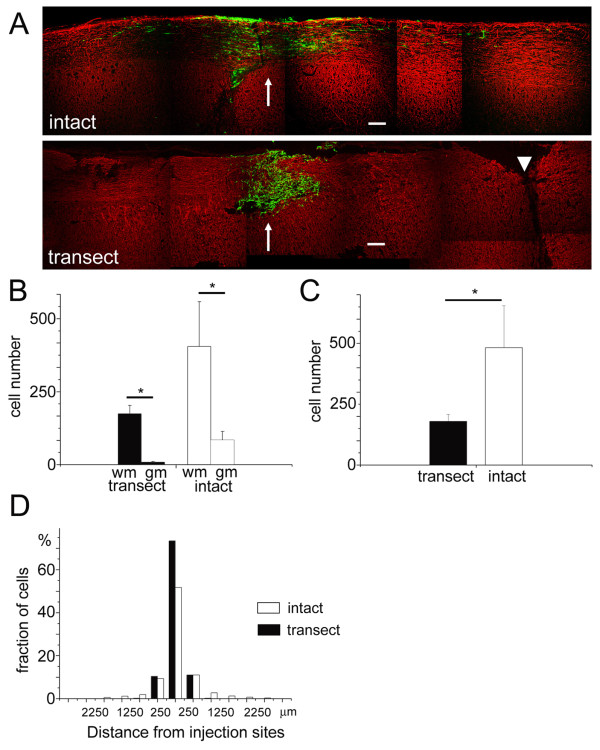
**Transplantation of NSPCs into the injured spinal cord**. (A) Montages of confocal microscopic images of the intact and injured spinal cords immunostained with anti-GFP antibody to detect the distribution of the transplanted NSPCs. The sites of spinal cord transection (arrowhead) are clearly separated from the sites of transplantation of NSPCs (arrows). The fixed spinal cord sections were stained with both anti-GFP antibody (green) and anti-neurofilament-200 antibody (red). Bar, 200 μm. (B) Numbers of surviving cells 7 days after transplantation in the white and gray matter. Single sections containing the highest number of GFP-positive cells were selected and the total numbers of surviving cells in these sections were taken as a representation of the extent of surviving cells. In both the intact and injured spinal cords, there was a preference of transplanted cells to reside within the white matter (intact gray matter; 83.4 ± 28.9 cells, intact white matter; 398.5 ± 151.5 cells, t-test p < 0.05, injured gray matter; 8.2 ± 2.8 cells, injured white matter; 171.5 ± 28.5 cells, t-test p < 0.05). (C) Numbers of surviving cells 7 days after transplantation in the total area of the spinal cord sections. The difference between injured and intact spinal cords was statistically significant (intact; 481.9 ± 173.3 cells, injured; 179.7 ± 28.6 cells, t-test p < 0.05). (D) Normalized rostrocaudal distribution of transplanted cells in the intact and injured spinal cords. The proportion of transplanted cell numbers in each 500 μm section along the rostrocaudal axis was plotted. The difference of the cell distribution was statistically significant (Mann-Whitney-U test, p < 0.05).

In this study, we first examined whether hippocampus-derived NSPCs show similar survival and migratory behavior in the injured spinal cord. We utilized complete transection of the neonatal spinal cord as a model of the spinal cord injury. In this injury model, the location and extent of the lesion can be precisely controlled. We generated complete transection of the spinal cord at the level of T9-10 on postnatal day 7. Transplantation of hippocampus-derived NSPCs expressing GFP was performed on postnatal day 14 at the level of T8-9. As a control, we introduced GFP-expressing NSPCs into the intact spinal cords of 14-day-old pups. Transduction of reporter genes by using recombinant adenoviruses was highly efficient, with more than 85% of NSPCs judged to be GFP-positive by fluorescence-activated cell sorting (FACS; data not shown). The extent of survival and migratory behavior in both groups 7 days after transplantation were analyzed by using immunofluorescence detection of transplanted cells with anti-GFP antibody staining (n = 6 for the injured spinal cord, n = 8 for the control). In both intact and injured environments, engrafted cells were detected at the original injection sites (Figure 1A and 1B). The cells at the injection sites were clearly separated from the sites of transection in all samples examined, indicating successful placement of NSPCs at the sites away from the transection level (Figure 1B). In both experimental conditions, transplanted cells were preferentially localized to the dorsal white matter (Figure 1B). We noticed less GFP-positive cells in the injured spinal cords and this observation was confirmed by counting the total number of transplanted cells (Figure 1C; control;481.9 ± 173.3 cells, injured; 179.7 ± 28.6 cells, mean ± SEM for all the data, t-test p < 0.05). The transplanted cells also showed less migratory behavior in the injured environment. Indeed, the distribution of GFP-positive cells in each 500 μm segment of the white matter along the rostrocaudal axis was statistically different and more transplanted cells were localized near the injection sites in the injured spinal cords (Figure 1D; Mann-Whitney-U test, P < 0.05). These results indicate an adverse environment of injured spinal cord for both survival and migration of hippocampus-derived NSPCs.

### Genetic manipulation of Rho family GTPases in NSPCs

The less favorable environment of the injured spinal cord will seriously impair neural tissue replacement therapy after spinal cord injury. One possible approach toward overcoming this difficulty is to genetically modify the donor NSPCs to be less vulnerable to the host environment. Toward this goal, we next evaluated the possibility of manipulating the activity of multiple Rho GTPases (RhoA. Rac1, and Cdc42) in NSPCs. Rho GTPases have been shown to be critically involved in both survival and migration of a variety of cell types. Therefore optimization of the levels of activities in Rho family GTPases can potentially influences the survival and migration of NSPCs in the context of spinal cord transplantation.

**Figure 2:**
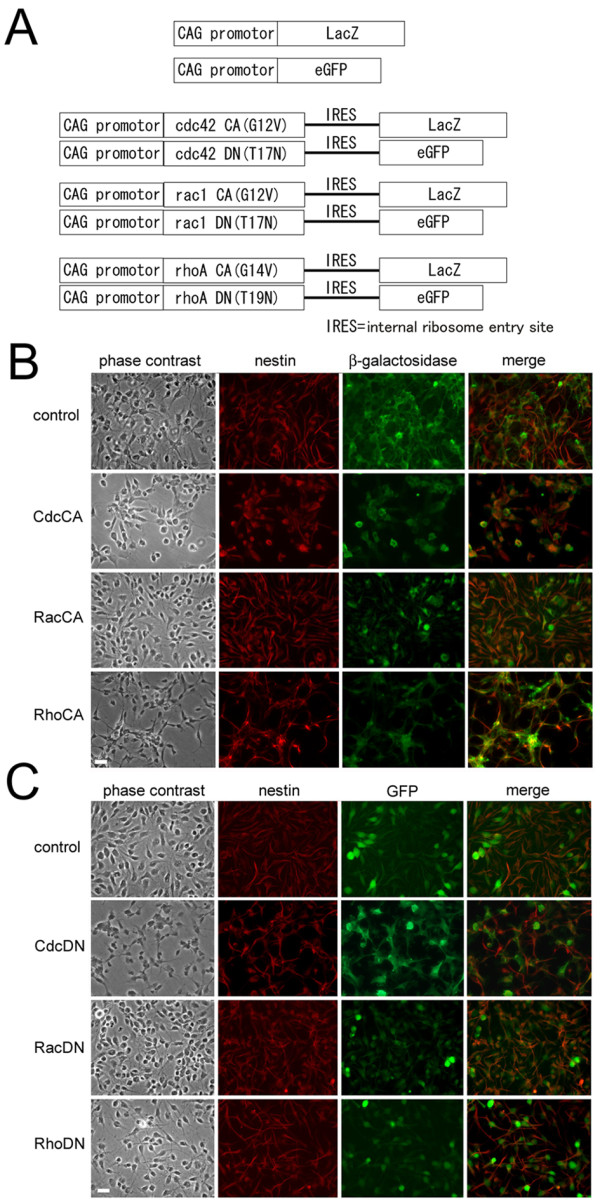
**Expression of mutated Rho GTPases in NSPCs**. (A) Design of expression constructs of recombinant adenoviruses. DNA fragments coding three CA forms of Rho GTPases (G12V mutant of cdc42, G12V mutant of rac1, G14V mutant of rhoA) were placed under the control of a CAG promoter in combination with the reporter gene lacZ. DNA fragments coding three DN forms of Rho GTPases (T17N mutant of cdc42, T17N mutant of rac1, T19N mutant of rhoA) were also placed under the control of a CAG promoter in combination with the reporter gene GFP. Intervening internal ribosomal entry sites (IRES) support the expression of reporter genes lacZ and GFP in these constructs. (B and C) Infection of cultured NSPCs with recombinant adenoviruses of CA forms (B) or DN forms (C) of Rho GTPases. Nestin-positive NSPCs expressed comparative amounts of β-galactosidase/GFP 2 days after infection. Bar, 20 μm.

NSPCs derived from rat embryonic hippocampus were maintained in the presence of bFGF. They were transfected with recombinant adenoviruses to express constitutive active (CA) or dominant negative (DN) forms of RhoA, Rac1, or Cdc42 under the control of the CAG promoter, along with marker genes (β-galactosidase for CA and GFP for DN, Figure 2A). Efficient expression of β-galactosidase or GFP in progenitors was observed two days after infection (Figure 2B and 2C). Immunoblotting using antibodies against RhoA, Rac1, or Cdc42 revealed overexpression of both CA and DN forms of individual Rho family GTPases in comparison with uninfected NSPCs (data not shown). We observed extensive overlap of cells immunopositive with neural stem cell marker nestin and cells expressing β-galactosidase or GFP, suggesting efficient gene transduction into the NSPC population (Figure 2B and 2C). Adenoviral infection itself did not have any deteriorative effects on both cell morphology and viability. To see if infected cells show normal differentiation phenotype, we quantified the numbers of cells immunopositive with anti-MAP2, anti-GFAP, and anti-RIP after differentiation of cells by bFGF withdrawal (Additional file 1). The fractions of differentiated cell types were not statistically different from those of control cells without recombinant adenovirus infection.

### Survival of NSPCs expressing Rho family GTPase mutants in the intact spinal cord

NSPCs genetically modified by recombinant adenoviruses expressing Rho GTPase mutants were grafted into intact spinal cords of 7 day-old rat pups. Data from 6 cases of control LacZ-expressing cells, 12 cases of control GFP-expressing cells, 5 cases of CdcCA-expressing cells, 11 cases of CdcDN-expressing cells, 12 cases of RacCA-expressing cells, 11 cases of RacDN-expressing cells, 10 cases of RhoCA-expressing cells, and 13 cases of RhoDN-expressing cells (80 cases in total) were analyzed in this study. We could detect donor cells in the host spinal cord 7 days after transplantation in all samples except those that received transplantation of cells expressing RhoCA. Absence of RhoCA-expressing donor cells in the intact spinal cord suggested negative effect of Rho activity in survival of transplanted NSPCs.

**Figure 3:**
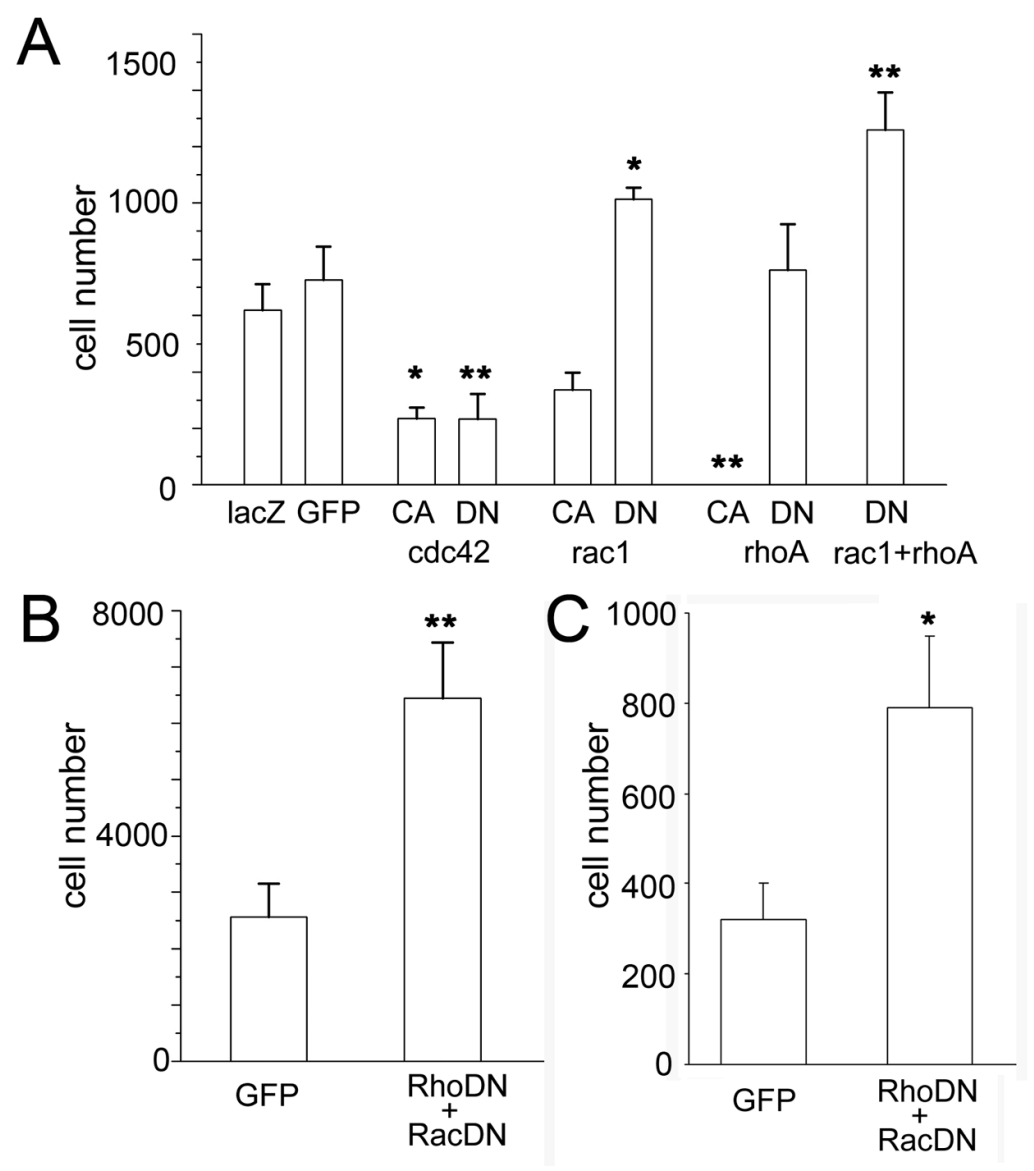
**Transplantation of NSPCs expressing mutant forms of Rho GTPases**. (A) Number of surviving cells in sections of spinal cords 7 days after transplantation. After transplantation of CA forms of cdc42 and rhoA, numbers of the surviving cells were significantly lower than the control cells expressing only lacZ (LacZ-positive control; 620.0 ± 90.9 cells, CdcCA; 235.6 ± 38.5 cells (p < 0.05), RacCA; 317.3 ± 60.5 cells, RhoCA; 0.0 cells (ANOVA, p < 0.01)). Although expression of the DN form of cdc42 reduced the number of surviving cells, expression of the DN form of rac1 increased the number of surviving cells (GFP-positive control; 723.3 ± 117.6 cells, CdcDN; 232.8 ± 88.8 cells (p < 0.01), RacDN; 1013.6 ± 39.7 cells (ANOVA, p < 0.05), RhoDN; 760.8 ± 162.9 cells). Double infection of adenoviruses expressing RacDN and RhoDN further enhanced the survival of transplanted NSPCs (1258.5 ± 133.6 cells (ANOVA, p < 0.01 in comparison with two types of control conditions)). (B) Increase in the total number of surviving NSPCs by expression of DN forms of both Rac and Rho. Complete serial sections of the transplanted spinal cords were examined and the total numbers of GFP-positive cells in two conditions were counted. A significant increase in surviving cells was observed in the condition of infecting two DN forms of Rho GTPases (control; 2565.2 ± 583.9 cells, RhoDN and RacDN; 6439 ± 990.6 cells, t-test p < 0.01). (C) Number of surviving cells in sections of spinal cords three weeks after transplantation. Double infection of adenoviruses expressing RacDN and RhoDN enhanced the survival of transplanted NSPCs (RacDN and RhoDN; 791.6 ± 156.9 cells, GFP-positive control; 320.8 ± 80.4 cells, t-test p < 0.05).

To more precisely evaluate the effects of manipulating the activity of Rho family GTPases, we measured the extent of cell survival by counting the numbers of transplanted cells marked with either β-galactosidase or GFP in representative histological sections containing the highest number of immunopositive cells (Figure 3A). Our previous analysis of transplanted NSPCs indicated little mitotic activity of NSPCs after transplantation. Therefore, a decrease in the number of cells surviving 7 days after transplantation mainly reflects the extent of cell death in the host environment. Transplantation of cells expressing β-galactosidase or GFP showed similar numbers of surviving cells, confirming similar detection sensitivity of the two markers and the reproducibility of our transplantation experiments. As already mentioned, we could not detect RhoCA-expressing cells in the sections, suggesting strong negative effects of RhoA activity in cell survival. Suppression of Rho activity by the DN construct, however, did not enhance donor cell survival, suggesting that the endogenous Rho activity in the transplanted cells does not play a major role in cell survival. Increase of Rac activity by RacCA results in similar, but milder reduction of the cell number. Importantly, suppression of Rac activity in transplanted cells by RacDN significantly enhanced the cell survival (139.4% of the control, ANOVA, p < 0.05). This result suggests the importance of endogenous activity of Rac in induction of cell death in NSPCs after transplantation. Cells expressing either CdcCA or CdcDN showed reduction in surviving cell numbers. Collectively, these results indicate differential effects of individual members of Rho family GTPases in survival of NSPCs after transplantation into the host spinal cord.

Down regulation of RhoA activity alone did not enhance survival of transplanted cells in our experiments. However, a previous report indicated that in the injured spinal cord, endogenous neurons and glial cells show Rho activation at the lesion sites and pharmacological prevention of Rho activation facilitates cell survival [19,21]. To further illustrate possible involvement of Rho activation in cell survival after transplantation, we generated NSPCs expressing both RacDN and RhoDN and characterized the survival of double positive cells 7 days after transplantation into the intact spinal cord. Microscopic examination and cell counting of representative single sections from each transplanted specimens revealed significant enhancement of cell survival (Figure 3A, ANOVA, p < 0.01). To more rigorously measure the extent of enhancement in cell survival, we counted the number of GFP-positive cells present in the complete sets of serial sections prepared from the spinal cords transplanted with either RacDN/RhoDN double positive cells (n = 5) or control cells expressing GFP (n = 5). On average, 6439 cells were present in all section (minimum number of cells (Nmin) = 3450, maximum number of cells (Nmax) = 9621) after transplantation of RacDN/RhoDN double positive NSPCs, and 2565 GFP-positive cells (Nmin = 434, Nmax = 3796) after transplantation of control cells (Figure 3B). However, the numbers of consecutive sections containing GFP-positive cells within the complete sets of serial sections were similar (8.2 sections on average with a range from 7 to 9 sections in transplantation of control cells, 7.8 sections on average with a range from 6 to 9 sections in transplantation experiments with RacDN/RhoDN double positive cells). Thus we obtained 2.5-fold increase of surviving cells after transplantation by modulating Rho family GTPase activity, albeit a similar extent of cell spreading in the host spinal cord.

Expression of both RacDN and RhoDN may facilitate long-term survival of transplanted NSPCs. To test this possibility, we analyzed the survival of transplanted NSPCs three weeks after transplantation. There were a large number of GFP-positive cells in the spinal cord three weeks after transplantation and the level of GFP expression and morphology of these cells was comparable to those one week after transplantation. The number of transplanted cells in single histological sections with the highest number of GFP-immunopositive cells was 791.6 ± 156.9 in the group of transplanting NSPCs expressing both RacDN and RhoDN, which was significantly higher than the value of the control (320.8 ± 80.4 cells per section, p < 0.05, Figure 3C). There was 66% decline of the surviving donor cells from one week to three weeks after transplantation in the control condition (from 481.9 ± 173.3 cells to 320.8 ± 80.4 cells). However, expression of both RacDN and RhoDN induced 2.5-fold increase of cell survival even at this later stage, indicating long-lasting effects of suppressing Rho family GTPases in the enhancement of cell survival after transplantation.

### Migration of NSPCs expressing Rho family GTPase mutants in the intact spinal cord

**Figure 4:**
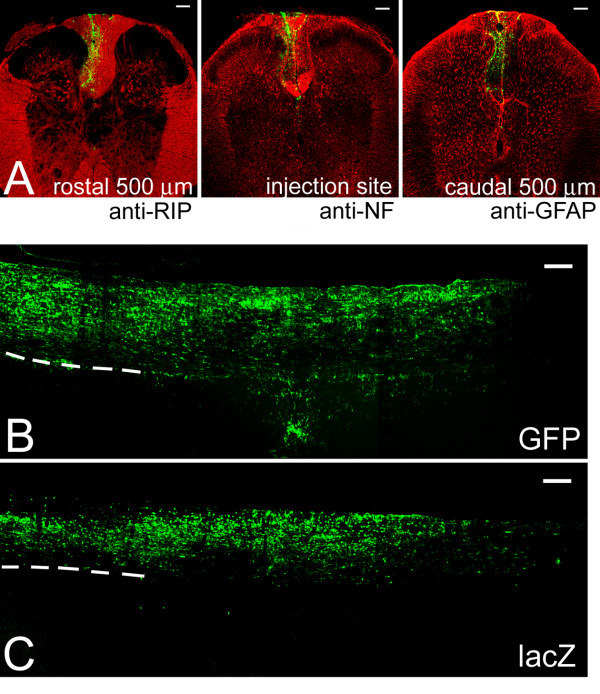
**Migration pattern of NSPCs expressing DN forms of both Rho and Rac after transplantation into the intact spinal cords**. (A) Distribution of the transplanted NSPCs expressing both RhoDN and RacDN at three different levels of the spinal cord. The sections were immunostained with specific markers for oligodendrocytes (RIP), neuronal axons (NF; neurofilament-200 antibody), and astrocytes (GFAP; anti-glial fibrillary acidic protein antibody). Transplanted cells are restricted to the dorsal white matter, where strong immunoreactivity of RIP and NFs was observed. Bar, 100 μm. (B and C) Rostrocaudal distribution of NSPCs expressing GFP (B) and LacZ (C) after spinal cord transplantation detected by immunostaining with anti-GFP antibody (B) or anti-β-galactosidase antibody (C). The dashed lines indicate the border between the white matter and the gray matter. Preferential localization of the immunoreactive cells in the white matter is evident. Bar, 200 μm.

Enhancement of cell survival by expressing RacDN and RhoDN is advantageous in neural tissue replacement therapy. However, migratory and differentiation phenotypes of the genetically manipulated cells should be examined to confirm the absence of abnormality. Microscopic evaluation on the distribution of transplanted cells revealed a similar pattern both in sagittal and coronal planes irrespective of the expressing Rho GTPase mutants except for RhoCA, which eliminated all the NSPCs 7 days after transplantation. Transplanted cells were distributed predominantly in the dorsal white matter and a small fraction of cells were detected in the gray matter. This result is consistent with our previous transplantation experiments of hippocampus-derived progenitor cells [20]. Cell migration from the injection sites was predominant along the rostrocaudal axis, and was restricted in both dorsoventral axis and mediolateral axis (Figure 4A, B, and 4C) in all cases of transplantation experiments.

**Figure 5:**
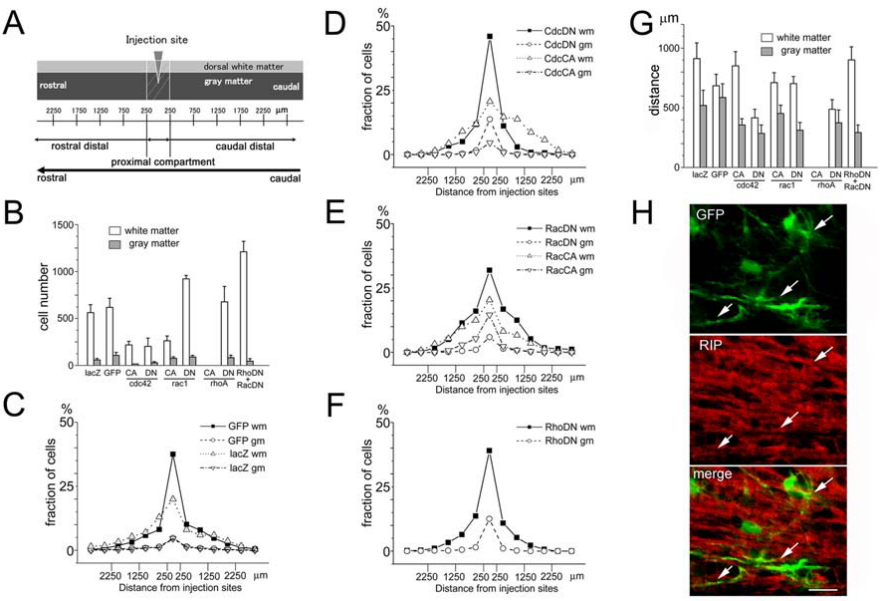
**Quantitative analysis of the migration pattern of NSPCs expressing mutant forms of Rho GTPases after transplantation**. (A) Schematic representation of the compartments defined by their distance from the injection site. Cells were grouped by their localization either within the white matter or the gray matter. Spinal cords were further subdivided into the proximal compartment, rostral distal compartment, and caudal distal compartment. The proximal compartment is the area within 250 μm from the injection sites. (B) Number of cells in the white matter or the gray matter. Preferential localization of transplanted cells in the white matter was observed in all NSPCs expressing CA or DN forms of Rho GTPases, except NSPCs expressing RhoCA. No surviving cells were detected in transplantation of NSPCs expressing RhoCA. (C-F) Migratory profiles of transplanted NSPCs expressing reporter genes (C) or mutant forms of Rho GTPases cdc42 (D), rac1 (E), and rhoA (F). In spite of the differences in the total number of surviving cells, the overall profiles of cell migration were similar in cells expressing mutant Rho GTPases. (G) Mean migratory distance of surviving NSPCs 7 days after transplantation. Cells expressing mutant forms of Rho GTPases showed a similar extent of migration along the rostrocaudal axis except for RhoCA. (H) Morphology and differentiated phenotype of transplanted cells expressing both RhoDN and RacDN. Ramified processes extended from the GFP-positive cell bodies and these structures were immunopositive with an oligodendrocyte marker RIP (arrows). Bar, 20 μm.

To further evaluate the migratory behavior, we divided the host spinal cord into 500 μm segment along the rostrocaudal axis, and counted the numbers of β-galactosidase or GFP-positive cells in each segments (Figure 5A). There was no significant difference in cell distribution among cells expressing different Rho GTPase mutants. In all cases examined, transplanted cells migrated into the dorsal white matter and the numbers of cells in the gray matter were significantly low (Figure 5B). We also plotted the profile of cell numbers along the rostrocaudal axis for cells expressing different Rho GTPase mutants. Qualitatively transplanted cells expressing different Rho GTPase mutants showed similar migratory profiles with their peak at the injection sites (Figure 5C-F). We then calculated the average migratory distances of transplanted cells from the injection sites. There was no significant difference in this parameter (Figure 5G), confirming an unaltered migratory phenotype of the cells with manipulated Rho GTPase activity.

We demonstrated previously that the transplanted NSPCs mainly differentiated into the oligodendroglial lineage [20]. A double-label immunofluorescence study with anti-GFP and anti-RIP (an oligodendrocyte marker) confirmed this trend in both control cells and cells expressing Rho GTPase mutants. Especially, both RacDN and RhoDN, which promoted survival of NSPCS, did not suppress differentiation of NSPCs into oligodendrocytes (Figure 5H). To estimate the extent of differentiation, we measured the proportion of RIP-positive cells in total GFP-positive cells and found no statistical difference between the control condition (76.9 ± 1.8%) and the condition of suppressing both Rac and Rho activities (76.2 ± 4.7%). The same proportions of CNPase -positive cells were confirmed as another oligodendrocyte marker, and the rest of RIP or CNPase positive cells in total GFP-positive cells were GFAP-positive cells. Quantitative analysis revealed that 20.7 ± 0.7% of GFP-positive cells was immunopositive for GFAP in control cells, and 23.7 ± 1.4% in RacDN and RhoDN cells. Neither control cells nor RacDN and RhoDN cells exhibited MAP2 expression in the host spinal cords. There is no difference between control cells and RacDN and RhoDN cells. Taken together, these results indicate unaltered migratory and differentiation capabilities of NSPCs after manipulation of Rho GTPase activity.

### Regulation of programmed cell death in vitro by manipulating Rho GTPase activity

**Figure 6:**
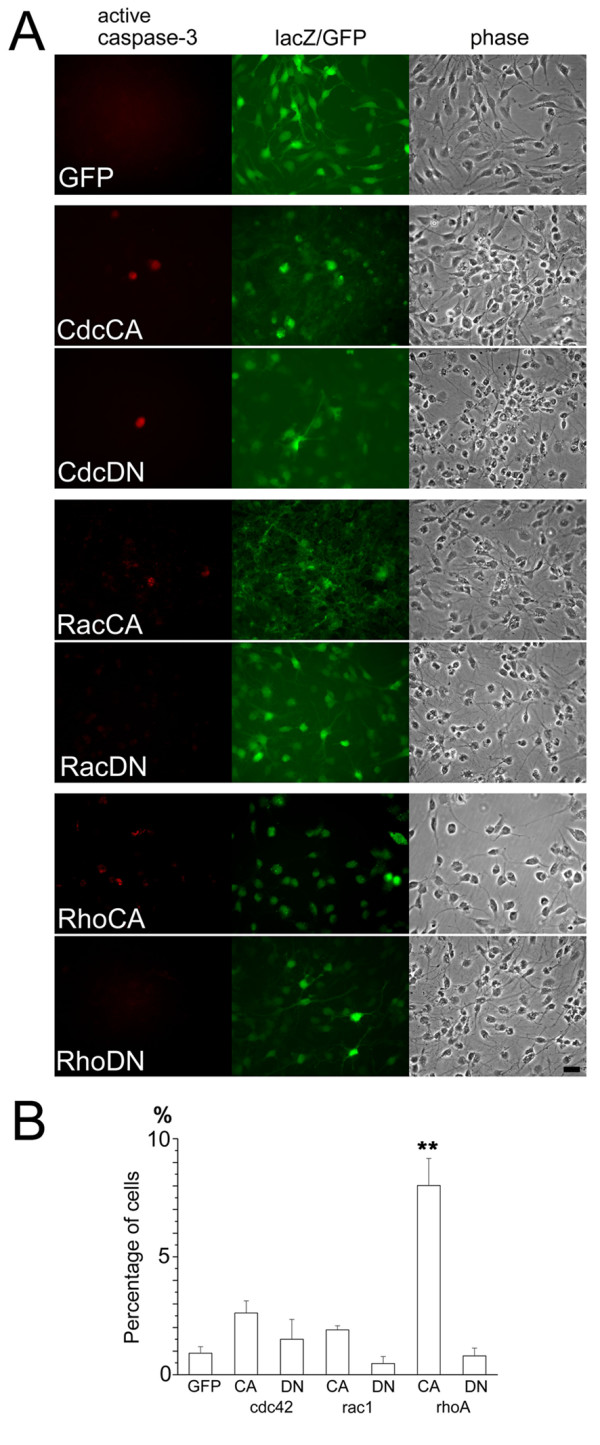
**Differential responses of NSPCs expressing mutated forms of Rho GTPases after withdrawal of bFGF**. (A) Anti-activated caspase-3 immunostaining of cultured NSPCs infected with recombinant adenoviruses expressing mutated forms of Rho GTPases. Activated caspase-3 immunoreactivity was observed in a small fraction of NSPCs (red). Similar extent of adenovirus infection was confirmed by immunostaining with anti-β-galactosidase antibody (green; CA mutations) or anti-GFP antibody (green; DN mutations). Bar, 20 μm. (B) Quantification of cells immunopositive with anti-activated caspase-3. Percentage of immunopositive cells is presented. There was a significant increase of cell death in cells expressing RhoCA (GFP control; 0.9 ± 0.3%, CdcCA; 2.6 ± 0.5%, CdcDN; 1.5 ± 0.6%, RacCA; 1.9 ± 0.2%, RacDN; 0.5 ± 0.3%, RhoCA; 8.0 ± 1,1% (ANOVA, p < 0.01), RhoDN; 0.8 ± 0.3%).

Both pro-apoptotic and pro-survival effects of Rho family GTPases have been reported in multiple types of cells in the neural lineages [16,22]. Our transplantation experiments suggested involvement of both Rho and Rac GTPase activity in inhibiting cell survival in the host environment. We next utilized an in vitro culture system of NSPCs to evaluate the roles of Rho GTPases in the process of programmed cell death induced by growth factor deprivation. Withdrawal of bFGF from the NSPC culture induced apoptosis in a small fraction of cells. Immunocytochemistry using an antibody against the cleaved form of caspase-3 detected the presence of activated caspase-3 in the cytoplasm of cells showing typical morphology of apoptotic cells. NSPCs genetically manipulated for their activity of Rho GTPases showed distinct responses to the withdrawal of bFGF (Figure 6A and 6B). Expression of RhoCA significantly increased the number of cells immunopositive with the activated caspase-3. Up-regulation of Cdc42 activity results in a slight increase of apoptotic cell number, which was not statistically significant. Both RhoDN and RacDN did not enhance cell death after withdrawal of bFGF. The overall pattern of promoting cell death by individual Rho GTPase mutants is reversibly correlated with the extent of cell survival after transplantation into the spinal cord (Figure 3A and Figure 6B), suggesting that the underlying molecular pathway is identical in two experimental paradigms.

**Figure 7:**
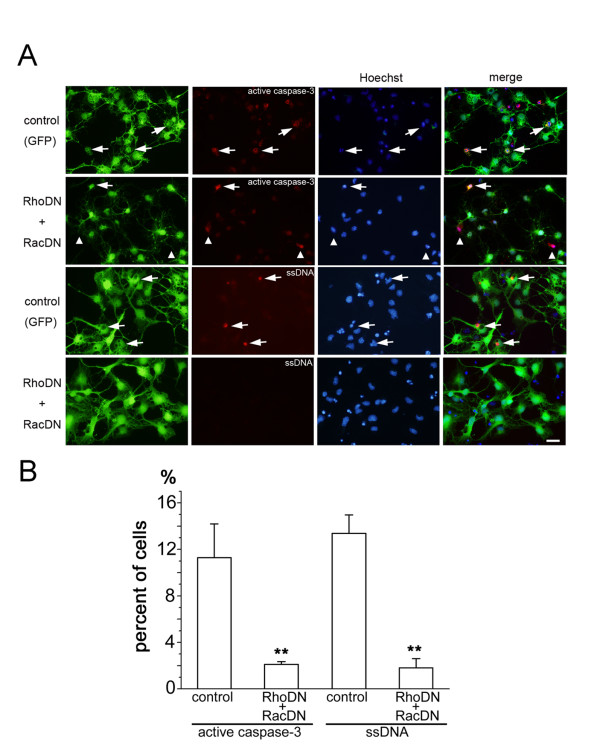
**Effects of suppressing Rho and Rac activity in staurosporine-induced cell death**. (A) Anti-activated caspase-3 and anti-ssDNA immunostaining of cultured NSPCs infected with recombinant adenoviruses expressing both RhoDN and RacDN. Higher proportions of anti-activated caspase-3 or ssDNA immunopositive cells (red) were observed in the control condition (arrows). Several GFP-negative cells expressed active caspase-3 (arrow heads). Cells were counterstained with Hoechst 33342 dye to visualize nuclei (blue). Bar, 20 μm. (B) Quantification of immunopositive cells for anti-activated caspase-3 or anti-ssDNA antibodies colocalized with GFP. Reduction of immunopositive cells was statistically significant (anti-activated caspase-3; p < 0.01, anti-ssDNA; p < 0.01, Mann-Whitney test).

Withdrawal of bFGF in NSPC cultures induced intrinsic apoptotic cascades in a small population of cells. To evaluate the effects of suppressing Rho and Rac GTPase activity in a condition of inducing more extensive cell death, we utilized the protein kinase inhibitor staurosporine. Apoptotic cells were identified by their nuclear morphology after staining with Hoechst dye, together with immunoreactivity against single-stranded-DNA (ssDNA) [23] or cleaved from of caspase-3. Application of staurosporine induced apoptosis in more than 10% of cells, which were also immunoreactive with antibodies against activated caspase-3 or ssDNA (Figure 7A). Suppression of both Rho and Rac activity by recombinant adenovirus infection reduced the induction of staurosporine-dependent apoptosis significantly (Figure 7B), suggesting strong pro-apoptotic effects of both Rho and Rac activities after a dramatic alteration of the cell environment, such as transplantation into the injured spinal cord.

### Enhancement of cell survival in the injured spinal cord by manipulating Rho GTPase activity

**Figure 8:**
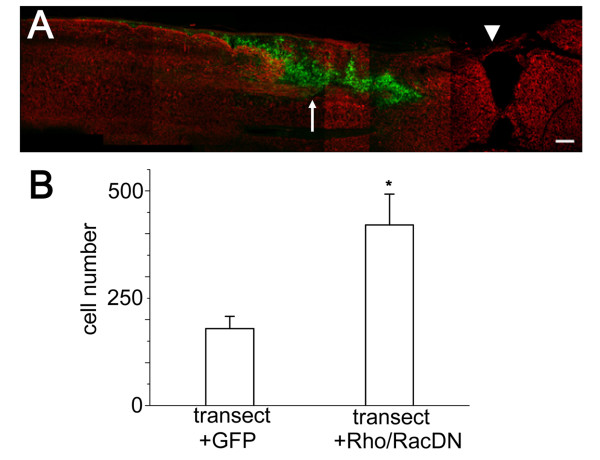
**Enhancement of cell survival in the injured spinal cord by suppression of Rho and Rac activity**. (A) Distribution of transplanted stem cells expressing both RhoDN and RacDN 7 days after transplantation. Cells expressing both RhoDN and RacDN (green) survived in the injured spinal cord (arrow). The sections were stained with anti-neurofilament-200 antibody (red). Arrowhead indicates the site of transection. Bar, 200 μm. (B) Quantification of cells present in the injured spinal cords 7 days after transplantation of NSPCs expressing RhoDN and RacDN. There was a significant increase of surviving cell numbers after transplantation of DN forms of Rho and Rac (control; 179.7 ± 28.6 cells (n = 6), RhoDN and RacDN; 420.6 ± 72.2 cells (n = 5), t-test p < 0.01).

Simultaneous suppression of Rac and Rho GTPase activities resulted in strong enhancement of cell survival both in the intact spinal cord after transplantation and in the culture system. We next analyzed if the manipulation of Rho family GTPase activity can enhance the survival of NSPCs in the injured spinal cord (Figure 8A and 8B). In control transplantation experiments, we identified 179.7 ± 28.6 GFP-positive cells (Nmin = 140, Nmax = 320) per single sections of the spinal cord. When NSPCs expressing both RacDN and RhoDN were transplanted, the number of surviving cells (420.6 ± 72.2) was 2.3-fold higher than the control condition and this difference was statistically significant (GFP; n= 6, DN; n = 5, t-test p < 0.01). To estimate the extent of differentiation, we measured the proportions of RIP- and GFAP-positive cells in total GFP-positive cells transplanted in the injured spinal cords. Quantitative analysis revealed that 23.3 ± 1.4%, 50.4 ± 2.6% of total GFP cells and 24.5 ± 1.6%, 54.7 ± 2.8% of the total RacDN and RhoDN cells were positive for RIP and GFAP, respectively. There is no difference between control cells and RacDN and RhoDN cells. Neither the control cells nor the RacDN and RhoDN cells exhibited MAP2 expression in the host spinal cords. Importantly, the number of surviving cells was comparable to that of transplanting wild-type cells into the intact spinal cord (Figure 1C and Figure 8B). Thus the genetic manipulation of Rho family GTPases can overcome the adverse host environment induced by the spinal cord injury.

## Discussion

In this study we identified Rho GTPase activity as a critical regulator of cell survival in the paradigm of NSPC transplantation into the rodent spinal cord. A less permissible environment for the NSPC created by spinal cord transection induced pronounced reduction in cell survival after transplantation. However, this reduction in cell survival was fully mitigated by controlling the activity of two Rho family GTPases, Rac and Rho.

We observed prominent reduction in the number of surviving cells after NSPC transplantation into the injured spinal cord. This adverse influence on cell survival can be derived from inflammatory response of the host cells. Tumor necrosis factor alpha (TNF-α) is a major cytokine produced acutely in the injured spinal cord. TNF-α induces demyelination and oligodendrocyte death in models of CNS injury [24,25]. Suppression of TNF-α production in injured spinal cord by the elevation of cAMP was shown to be correlated with functional recovery [26]. However, a previous study demonstrated rapid down-regulation of TNF-α transcripts in the injured spinal cord, suggesting a less prominent role of TNF-α one week after injury, when the expression level of TNF-a returned to the level of the non-operated control tissue [27]. Another candidate preventing NSPC survival is p75^NTR^, which is also a member of the TNF receptor family. In addition to its function as a low-affinity receptor for NGF, p75^NTR ^forms a receptor complex together with Nogo-66 receptor and LINGO-1, transmitting the signal from myelin-derived inhibitors [28-30]. Rho directly binds p75^NTR ^and this interaction modulates Rho activity [31,32]. Activation of Rac by p75^NTR ^induces apoptosis via activation of JNK in oligodendrocytes [18]. It is possible that up regulation of three members of the TNF receptor family in the injured spinal cord is involved in the apoptotic cell death of transplanted NSPCs and this process can be modulated by the activity of Rho GTPases.

Rho GTPases have diverse functions in the neural tissue. Several lines of evidence indicate that Rho GTPases regulate cell survival. Rac/Cdc42 GTPases promote the apoptotic death of NGF-deprived sympathetic neurons [16,17] and activation of Rac by p75^NTR ^induces apoptosis via activation of JNK [18]. These studies suggest negative roles of Rho family GTPases in cell survival. However, in the case of cerebellar granule cells, down-regulation of Rac GTPase induces apoptosis through the c-Jun-dependent induction of the BH3-only protein Bim and the subsequent activation of a mitochondrial apoptotic cascade [22]. These previous reports indicate that the balance between pro-survival and pro-apoptotic effects of Rho GTPase is cell-type and context dependent. Our in vitro data indicate strong effect of Rho GTPase up regulation on the enhancement of an apoptotic cascade after withdrawal of bFGF. Furthermore, suppression of both Rho and Rac GTPase activity by DN constructs resulted in strong inhibition of a mitochondrial apoptotic cascade induced by staurosporine. These observations support the view that activation of Rho and Rac in NSPCs both in vitro and in the injured spinal cord accelerates activation of an apoptotic cascade. DN and CA forms of Rho family GTPase mutants have been widely used to specifically regulate signaling pathways mediated by Rho, Rac or Cdc42 [33]. However, it has also been reported that dominant-negative mutants of Rho family GTPases compete for the same guanine nucleotide exchange factors, and therefore their inhibitory effects do not reflect the phenotype of selective inactivation [34]. We observed differential effects of three DN mutants on the survival of transplanted NSPCs in a condition with a similar level of adenoviral infection, suggesting pathway-specific effects of these DN mutants. At least, opposite effects of CdcDN and RacDN on the survival of transplanted cells can not be explained by non-specific competition of guanine nucleotide exchange factors by multiple DN mutants. Negative effect of both CdcDN and CdcCA on the survival of transplanted cells may reflect the importance of controlled cycling of Cdc42 between an active GTP-bound and inactive GDP-bound state for proper regulation of cell proliferation and survival. This property is unique to Cdc42 mutants, further supporting selectivity of individual DN and CA mutants.

Another important function of Rho family GTPases is regulation of cell motility. In fibroblasts, Rho regulates the formation of contractile actin-myosin filaments to form stress fibers, while Rac and Cdc42 regulate lamellipodia and filopodia formation, respectively [14]. In developing neurons, Rho activity is important in the growth cone motility [35,36]. Chemotaxis in a variety of cell types is regulated by local activation of Rac or Ras, and subsequent establishment of a phosphoinositide gradient by phosphatidylinositol 3'-kinase (PI3K) is also important [37,38]. In this sense, we expected to see profound effects of manipulating Rho GTPases on the distribution of transplanted cells in the spinal cord. However, there was no significant difference in the migration pattern of transplanted cells along the rostrocaudal axis and also in their preferential accumulation in the dorsal white matter. Motile behavior of neural precursor cells during normal brain development has been studied extensively using real-time imaging of both slice preparations and in vivo imaging [39-41]. In turn, characterization of NSPC motility in the injured tissue environment is not yet fully characterized. A recent report indicates the importance of chondroitin sulfate proteoglycans (CSPGs) as a putative negative regulator of NSPC migration [42]. It is not yet clear, however, if this regulatory effect of CSPGs is mediated by Rho GTPase-dependent signaling pathway.

It is possible that the migration of transplanted NSPCs in the spinal cord is independent of Rho GTPase activation and its control on the actin reorganization. In this sense, the effects of other regulators of actin dynamics on the migratory behavior of transplanted cells should be intriguing [38]. It is also important to perform real-time analysis of cell migration and dynamic morphological change after transplantation, either by microscope-based imaging [43] or bioluminescence-based whole body-imaging [44].

Rho GTPases are also involved in the maintenance of stem cells and their directed differentiation. In the case of hematopoietic stem/progenitor cells (HSC/Ps), genetic deletion of Rac1 in HSC/Ps results in their reduced ability to reconstitute hematopoiesis in vivo [45-47]. Rac2 null HSC/Ps displayed different phenotype, indicating distinct roles of two Rac isoforms in hematopoietic development. Rac1 is also essential in the maintenance of stem cells in the epidermis [48]. Conditional knockout of Rac1 in the epidermis resulted in depletion of stem cell population and this effect is dependent on negative regulation of c-Myc activity through p21-activiated kinase 2. Rho activity is involved in the fate decision of mesenchymal precursor cells. Mesenchymal precursor cells can differentiate into both adipocytes and myocytes by stimulation with insulin-like growth factor-1. Activity level of Rho GTPase is a component of a critical switch in this fate decision [49]. Similar regulatory roles of RhoA have been reported in the case of fate decision between adipocytes and osteoblasts from mesenchymal precursors [50]. Do Rho GTPases influence the maintenance of stem cell state and subsequent fate decision in NSPCs? We observed an unaltered differentiation phenotype of NSPCs expressing Rho GTPase mutants after transplantation and also after induction of in vitro differentiation by growth factor withdrawal. Proliferation of NSPCs in the presence of bFGF also did not show prominent change by expressing Rho GTPase mutants. In addition, our previous transplantation experiments in combination with subsequent administration of 5-Bromo-2-deoxyuridine (BrDU) indicated that proliferation of NSPCs did not take place in the host spinal cord [20]. These observations collectively indicate minimal impacts of manipulating Rho GTPase activity on proliferation/differentiation of NSPCs after transplantation.

It has been reported that transplanted neural stem cells originated from E14 cerebral cortex were differentiated preferentially into astrocytes in adult intact and injured spinal cords [51]. They also showed that the number of differentiated astrocytes increased during the observation period after injury. In our study, the proportion of astrocyte differentiation from the transplanted NSPCs is higher in the injured than in the intact spinal cords. The injured environments may provide a field with increased factors which induce astrocyte differentiation, such as CNTF and IL-6 [52,53]. Recently, it is also reported that elevation of both FGF2 and CNTF level after spinal cord injury induces development of oligodendrocytes [54]. Previously, we indicated that higher number of transplanted NSPCs differentiated into oligodendrocytes expressing RIP or CNPase in the intact neonatal spinal cords [20]. In embryonic and neonatal stages, the Notch-signaling plays an important role in differentiation of oligodendrocytes [55]. The transplanted NSPCs can be affected by controlling the Notch level under in intact neonatal spinal cords. These reports may explain our observation that NSPCs preferentially differentiate into oligodendrocytes in the intact, but into astrocytes in the injured spinal cords. Artificially preventing differentiation of transplanted NSPCs into astrocytes by controlling injured environments may make more oligodendrocytes which should facilitate axonal regeneration after spinal cord injury.

## Conclusion

In vitro-expanded NSPCs derived from the fetal hippocampus are ones of the potential sources for transplantation therapy of the damaged CNS including the spinal cord. Throughout characterization of both cell-autonomous and environmental factors influencing survival, migration, and differentiation of NSPCs should be critical in designing the optimal strategy to repair the injured CNS. Manipulation of factors intrinsic to the NSPCs, such as the activity of signaling molecules characterized in this study, is a promising approach, as recent development of genetic manipulations enabled us to activate or inactivate individual components of diverse signaling cascades precisely.

## Methods

### FGF-responsive NSPC culture

The generation and characterization of embryonic neural stem cell cultures were described previously [3,20]. In brief, the hippocampi of E16 embryos from pregnant Sprague-Dawley rats were dissected in Ca^2+^- and Mg^2+^- free Hanks' Balanced Salt Solution (HBSS) supplemented with 16 mM Na-HEPES (pH 7.2), and mechanically dissociated. The cells were collected and resuspended in N2 medium [56] and bFGF (10 ng/ml; R&D systems, Minneapolis, MN). Cells were grown on a 10-cm plastic dish precoated with poly-L-ornithine (Sigma, St. Louis, MO) and fibronectin (Invitrogen, Carlsbad, CA). bFGF was added daily, and the medium was replaced every 2 days. Cells were maintained for 7 days after initial plating. The cells were passaged at 60-70% confluence by brief incubation in Ca^2+^- and Mg^2+^- free HBSS and dislodging with a cell lifter. To induce staurosporine-dependent apoptosis, NSPCs passaged once and maintained for additional 4-5 days in the presence of bFGF were exposed to staurosporine (0.25 mM) for 18 h [57].

### Recombinant adenoviruses-mediated gene transfer

Recombinant adenoviruses expressing constitutive active (CA) or dominant negative (DN) forms of Cdc42, Rac1 or RhoA, together with marker genes (lacZ for CA and GFP for DN) under the control of a CAG promoter were constructed as previously described using Adenovirus Expression Vector Kit (Code 6150: TaKaRa New York, NY) [58]. Titers of adenoviruses were as follows; Ad-GFP; 5.0 × 10^9^, Ad-LacZ; 4.3 × 10^9^, cdc CA; G12V2.3 × 10^10^, cdc DN T17N; 1.7 × 10^10^, rac CA G12V; 3.3 × 10^9^, rac DN T17N; 6.76 × 10^10^, rho CA G14V; 5.1 × 10^9^, rho DN T19N; 6.7 × 10^10 ^(PFU/ml). NSPCs were infected with concentrated stocks of adenoviruses after adjustment of their final PFUs. By using the internal ribosome entry site (IRES)-based bicistonic vector system, both marker genes and mutant forms of Rho GTPases were expressed by infection of single adenoviruses. NSPCs maintained in the presence of bFGF for 6 days after the initial passage were exposed to recombinant adenoviruses for 2 days. The cells exposed to recombinant adenoviruses were washed three times with HBSS, mechanically dissociated, collected by brief centrifugation and resuspended in N2 medium at a concentration of 10^5 ^cells/μl.

### Immunocytochemistry of cultured NSPCs

NSPCs exposed to recombinant adenoviruses containing lacZ or GFP reporter genes were fixed with 2% paraformaldehyde (PFA) in PBS for 30 min. After treatment with 0.2% Triton X-100 for 5 min, cells were blocked with 5% NGS and incubated with anti-nestin antibody (Developmental Studies Hybridoma Bank, Iowa City, IA). Primary antibody was visualized with goat anti-mouse IgG conjugated to Cy3 (1:200, Jackson ImmunoResearch, West Grove, PA). LacZ positive cells were subsequently stained by using rabbit polyclonal anti-β-galactosidase antibody (ICN, Costa Mesa, CA) and secondary antibody conjugated to Alexa 488 (Molecular Probes, Eugene, OR). In the experiments where cells were manipulated to induce apoptotic cell death [57], anti-activated caspase3 antibody (Promega, Madison, WI) and anti-single stranded DNA (ssDNA) antibody (DAKO, Glostrup, Denmark) were used as the primary antibody and both were visualized with goat anti-rabbit IgG conjugated to Cy3. To evaluate effects of adenovirus-mediated expression of CA or DN forms of Rho family GTPases on differentiation of NSPCs in vitro, subconfluent NSPCs were exposed to recombinant adenoviruses, maintained in the presence of bFGF for two days, and then switched to the differentiation condition without bFGF for the following two days. The cells were fixed with 2% PFA in PBS, and stained with microtubule associated protein 2 (MAP2; Sigma), glial fibrillary acidic protein (GFAP; Sigma), or RIP (Developmental Studies Hybridoma Bank) to determine their differentiation phenotype. The quantification of cells was done by capturing images of cells immunopositive for neuronal/glial markers, together with Hoechst33342 (Molecular Probes) staining as a nonselective nuclear marker, in randomly selected five areas within a dish. In quantitative analysis, 40-121 cells were counted per each area, with total numbers of cells in the range of 200-644. More than two independent culture preparations were analyzed and averaged.

### Animals and surgical procedures

The animal care committee of Tokyo Medical and Dental University approved all animal experiments. Neural progenitor cells expanded in vitro were genetically labeled with recombinant adenoviruses and grafted into intact or previously transected spinal cords of 14-day-old rat pups. For transplantation of NSPCs, animals were cryoanaesthetized and laminectomy (at the level of T9-10 for intact spinal cords and T8-9 for transected spinal cords) was performed. The dura was cut, and 1 μl of the cell suspension (10^5 ^cells per μl) was gently injected into the right side of the gray matter using a glass micropipette (tip diameter; 100 μm) at a depth of less than 1 mm. The glass micropipette was held in place for 2 min and slowly withdrawn. Complete transection of the spinal cord at the level of T9-10 was performed by using an ophthalmic blade on 7-day-old rat pups. In the data set for the comparison of intact and transected spinal cords without manipulation of Rho family GTPases, we analyzed data from 6 successful transplantations into injured spinal cords (6 out of 8 trials), and 8 transplantation into uninjured spinal cords (8 out of 10 trials). In the data set for the short-term manipulation of Rho family GTPase activity, 80 transplantation experiments were performed on 7-day-old rat pups with one week interval between transplantation and sample fixation (LacZ: n = 6, GFP: n = 12, cdcCA: n = 5, cdcDN: n = 11, racCA: n = 12, racDN: n = 11, rhoCA: n = 10, rhoDN: n = 13). For the analysis of long-term effects of manipulating Rho family GTPase, the NSPCs expressing either GFP alone (n = 5) or the combination of two DN mutants (racDN pus rhoDN; n = 5) were transplanted into spinal cords of 7-day-old rat pups and maintained three weeks before analysis.

### Immunohistochemistry of transplanted spinal cords

To characterize migration pattern and differentiation of transplanted NSPCs in the host spinal cord, immunohistochemistry using anti-β-galactosidase antibody or anti-GFP antibody (mouse monoclonal, gift from Dr. S. Mitani; rabbit polyclonal, Molecular Probes, Eugene, OR) was performed. One or three weeks after transplantation, animals were intracardially perfused by 2% PFA in PBS under terminal chloral hydrate anesthesia (600 mg/kg body weight, intraperitoneal injection). In the transplantation experiments without spinal cord transection, the spinal cords were carefully isolated and processed with microslicer (Dosaka EM, Kyoto, Japan)to obtain sagittal or axial sections (50 μm). In the transplantation experiments into the injured spinal cords, fixed spinal cords were dissected, cryoprotected with sucrose and embedded in 1% agar with PBS for further processing with a cryostat to produce sagittal sections (20 μm). Selected sections were stained by using anti-β-galactosidase or anti-GFP antibody and secondary antibody conjugated to Alexa 488 for the detection of transplanted cells. To detect colocalization with various neural markers, the sections were further processed by reacting with either anti-MAP2 (Sigma), neurofilament-200 (Sigma), anti-GFAP (Sigma), anti-CNPase (Sigma), or RIP (Developmental Studies Hybridoma Bank) antibodies and subsequently incubated with secondary antibodies conjugated to Cy3. Images were obtained on a Fluoview confocal laser microscope (Olympus, Tokyo, Japan).

### Image analysis

To quantitatively evaluate the migratory behavior of transplanted cells, we serially sectioned spinal cords and examined anti-GFP- or anti-β-galactosidase immunopositive cells by fluorescence microscope with a low magnification objective lens (X10). We selected a single section that contained the highest number of GFP- or β-galactosidase-positive cells. In experiments of survival of GFP-positive control cells and RacDN/RhoDN double positive cells, we analyzed the complete sets of serial sections. Each 500 μm segment was examined with a high magnification objective lens (X60) to identify single cells and their number per segment. Only GFP- or β-galactosidase positive cells which showed simultaneous labeling of nuclei with Hoechst nuclear dye were counted. This procedure reduces the possibility of double counting same cells in adjacent sections. We first calculated the sum of cell numbers in 500 μm segments to obtain total number of cells per microslicer section and then summed the total number of cells per section to obtain the total number of transplanted cells per spinal cord. As we did not attempt to incorporate stereological techniques, sampling bias is likely to be present in cell number estimation. However, this sampling bias will affect the cell number estimation of transplanted cells both in control and manipulated spinal cords proportionally. Therefore we did not attempt to incorporated stereological techniques to obtain corrected total number of transplanted cells.

In experiments of evaluating migratory behavior of transplanted cells, we selected a single section which contained the highest number of GFP- or β-galactosidase-immunopositive cells among serial sections. In most cases, alignment of sections with the rostrocaudal axis was good enough to hold both the most rostral and caudal part of the spinal cord where transplanted cells existed. When the alignment of sections was not good enough to reveal the entire profile of cell distribution in a single section, these samples were excluded from the analysis. In experiments with normal spinal cords, the position of needle placement was identified by the presence of scar tissue and this position (injection site) was set to the center of initial 500 μm segment (Figure 5A). In experiments with injured spinal cords, the position of spinal cord transection was also recorded in addition to the injection site. Toward both rostral and caudal part of the spinal cord, multiple 500 μm segments were set sequentially, with the initial segment at the injection site as a reference of the central segment. GFP- or β-galactosidase-immunopositive cells were detected by using a high magnification objective lens (X60) and their total number per segment and the numbers of cells in either the dorsal white matter or the gray matter were counted. The average migratory distance of transplanted cells (L) in individual sections of the spinal cord was calculated by using the following equation;

Where n = number of cells present in each 500 μm segment, y = distance of the center of each segment from the injection site, N = total cell number present in the section.

## List of abbreviations

bFGF: basic fibroblast growth factor; CA: constitutive active form; CNS: central nervous system; DN: dominant negative form; GFP: green fluorescent protein; JNK: c-jun N-terminal kinase; NSPC: neural stem/progenitor cell; p75NTR: p75 neurotrophin receptor; ssDNA: single stranded DNA; TNF: tumor necrosis factor.

## Competing interests

The authors declare that they have no competing interests.

## Authors' contributions

FN designed and conducted all experiments. AK conducted data analysis and prepared the manuscript. ME conducted animal surgery, immunochemistry and statistical analysis. KS and AO contributed to project development and provided critical review and comments. SO supervised the project and wrote the manuscript. All authors read and approved the final manuscript.

## Supplementary Material

Additional file 1 **The proportion of in vitro differentiation in each adenovirus infected cell**. The immunocytochemical analysis was performed for in vitro-expanded NSPCs three days after withdrawal of bFGF. There were no significant differences between the phenotypes of NSPCs infected with recombinant adenoviruses and noninfected control cells. Click here for file
