# Supplementary material for: Critical involvement of Rho GTPase activity in the efficient transplantation of neural stem cells into the injured spinal cord
Source: Mol Brain. 2009 Nov 28;2:37. doi: 10.1186/1756-6606-2-37 (PMC2789715; doi:10.1186/1756-6606-2-37)
Supplement: Additional file 1 — The proportion of in vitro differentiation in each adenovirus infected cell. The immunocytochemical analysis was performed for in vitro-expanded NSPCs three days after withdrawal of bFGF. There were no significant differences between the phenotypes of NSPCs infected with recombinant adenoviruses and noninfected control cells. [file 1756-6606-2-37-S1.pdf]

Additional file 1. The proportion of in vitro differentation in each adenovirus infected cell

	control	CdcCA	CdcDN	RacCA	RacDN	RhoCA	RhoDN	RacDN+RhoDN
MAP2	19.5±1.5	21.0±1.8	20.4±1.3	21.2±2.7	20.5±1.7	21.8±1.0	22.9±2.3	23.7±1.7
GFAP	13.6±1.6	16.3±2.0	15.2±0.3	15.0±1.6	14.2±1.1	13.3±1.0	13.4±0.8	16.1±0.8
RIP	3.2±0.3	2.8±0.3	2.9±0.3	3.7±0.3	4.2±0.5	3.5±0.3	4.6±1.5	4.9±0.7

(% of counted cells ± SEM)
